# Recessive myotonia congenita caused by a homozygous splice site variant in *CLCN1* gene: a case report

**DOI:** 10.1186/s12881-020-01128-5

**Published:** 2020-10-22

**Authors:** Peter Sparber, Margarita Sharova, Alexandra Filatova, Olga Shchagina, Evgeniya Ivanova, Elena Dadali, Mikhail Skoblov

**Affiliations:** Research Centre for Medical Genetics Moskvorechie 1, Moscow, 115522 Russia

**Keywords:** Myotonia congenita, Becker disease, Functional analysis, Splicing, Case report

## Abstract

**Background:**

Myotonia congenita is a rare neuromuscular disease, which is characterized by a delay in muscle relaxation after evoked or voluntary contraction. Myotonia congenita can be inherited in a dominant (Thomsen disease) and recessive form (Becker disease) and both are caused by pathogenic variants in the *CLCN1* gene. Noncanonical splice site variants are often classified as variants of uncertain significance, due to insufficient accuracy of splice-predicting tools. Functional analysis using minigene plasmids is widely used in such cases. Moreover, functional analysis is very useful in investigation of the disease pathogenesis, which is necessary for development of future therapeutic approaches. To our knowledge only one noncanonical splice site variant in the *CLCN1* gene was functionally characterized to date. We further contribute to this field by evaluation the molecular mechanism of splicing alteration caused by the c.1582 + 5G > A in a homozygous state.

**Case presentation:**

We report a clinical case of an affected 6-y.o boy with athletic appearance due to muscle hypertrophy, calf muscle stiffness, cramping and various myotonic signs in a consanguineous family with no history of neuromuscular disorders. The neurological examination showed percussion-activated myotonia in the hands and legs. Plasma creatine kinase enzyme and transaminases levels were normal. Electromyography at the time of examination shows myotonic runs in the upper and lower extremities.

**Conclusions:**

Functional analysis of the variant in a minigene system showed alteration of splicing leading to loss of function, thereby confirming that the variant is pathogenic.

## Background

Myotonia congenita (MC) belongs to the group of non-dystrophic myotonias along with paramyotonia and sodium channel myotonia. Clinically myotonia defined by muscle stiffness in various muscle groups, percussion myotonia, and the “warm-up” phenomenon (relieve in myotonia after repetitive contractions) with myotonic discharges seen on electromyography (EMG) [1]. MC is associated with pathogenic variants in the *CLCN1* gene, which encode for the skeletal muscle chloride channel CLC-1 and can be inherited in a dominant or recessive trait. The recessive form of MC (Becker disease – OMIM #255700) typically has a more severe phenotype and is considered to be more widespread than the dominant one (Thomsen disease – OMIM #160800) [2].

The overall prevalence of MC is 1:100′000 according to the Orphanet database [3]. However the prevalence of MC can be as high as 1:10′000 in Northern Finland [4]. The disease onset of recessive MC is usually in the first or the second decade of life starting with painless myotonia in the lower limbs and progresses to the arms, facial muscles and neck. Patients can have an athletic appearance due to muscle hypertrophy. Transient muscular weakness is also a common feature.

We are reporting a clinical case of a Turkmen consanguineous family where a 6-y.o boy at the time of examination had calf muscle stiffness with periodic muscle spasms and various myotonic phenomena’s without signs of muscle dystrophy. Sanger sequencing of the *CLCN1* gene identified a homozygous variant at the intron 14 - c.1582 + 5G > A. Functional analysis in a minigene system was performed in order to evaluate the impact of the variant on splicing.

## Case presentation

A family of Turkmen origin was referred to genetic consultation for progeny prediction. One of their three children - a 6-year old boy was examined due to calf muscle stiffness and cramping in various muscle groups. The proband was born in consanguineous marriage - his parents were third cousins. Gestation and delivery were uneventful. He developed normally for the first year of life when painless muscle stiffness of the calf muscle was first noticed. The parents had two healthy daughters without any neurological signs. No history of neuromuscular disorders was present in the family.

Upon examination at the age of six, the proband had an athletic appearance due to muscle hypertrophy. The neurological examination showed percussion-activated myotonia in the hands and legs. After a maximum voluntary contraction of the hand, the proband could not fully open his fist, but repeated attempts improve myotonia due to the warm-up phenomenon. Plasma creatine kinase enzyme and transaminases levels were normal. Electromyography shows myotonic runs in biceps brachii, vastus lateralis and tibialis anterior muscles.

**Fig. 1:**
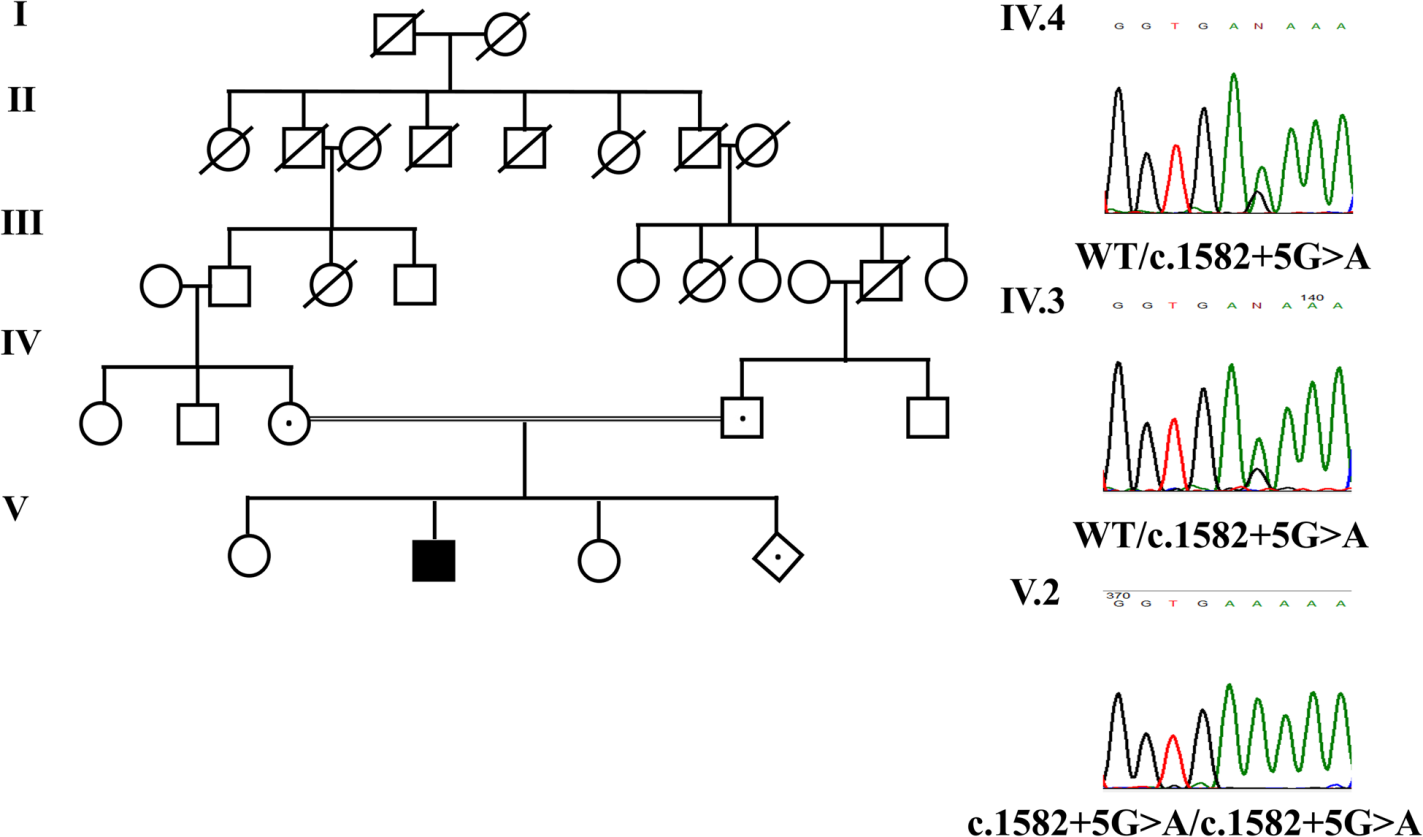
Left panel shows the pedigree of the affected family. Black symbol indicate affected individual. Black dotes indicated unaffected carriers – the probands parents and the fetus. Right panel shows the electropherograms of the identified variant in the proband and his parents

Based on the clinical findings a recessive form of MC was suspected. Sanger sequencing of all *CLCN1* exons and the nearest intron regions was performed. It revealed a homozygous variant in the intron 14 of the *CLCN1* gene - c.1582 + 5G > A. Following segregation analysis in the family showed that both parents are heterozygous for this variant (Fig.1).

Homozygous or compound heterozygous pathogenic variants in the *CLCN1* gene lead to recessive MC also known as Becker disease. c.1582 + 5G > A variant was described as pathogenic in a previous work [5]. However, no functional studies were made to confirm the functional consequences of the discovered variant and according to ACMG guidelines this variant should be classified as a “variant of uncertain significances” (VUS) [6]. This means that we cannot be sure about the pathogenic role of the described variant and his causality. In silico analysis using Human Splicing finder 3.1 [7] predicted a broken wild-type donor site.

**Fig. 2:**
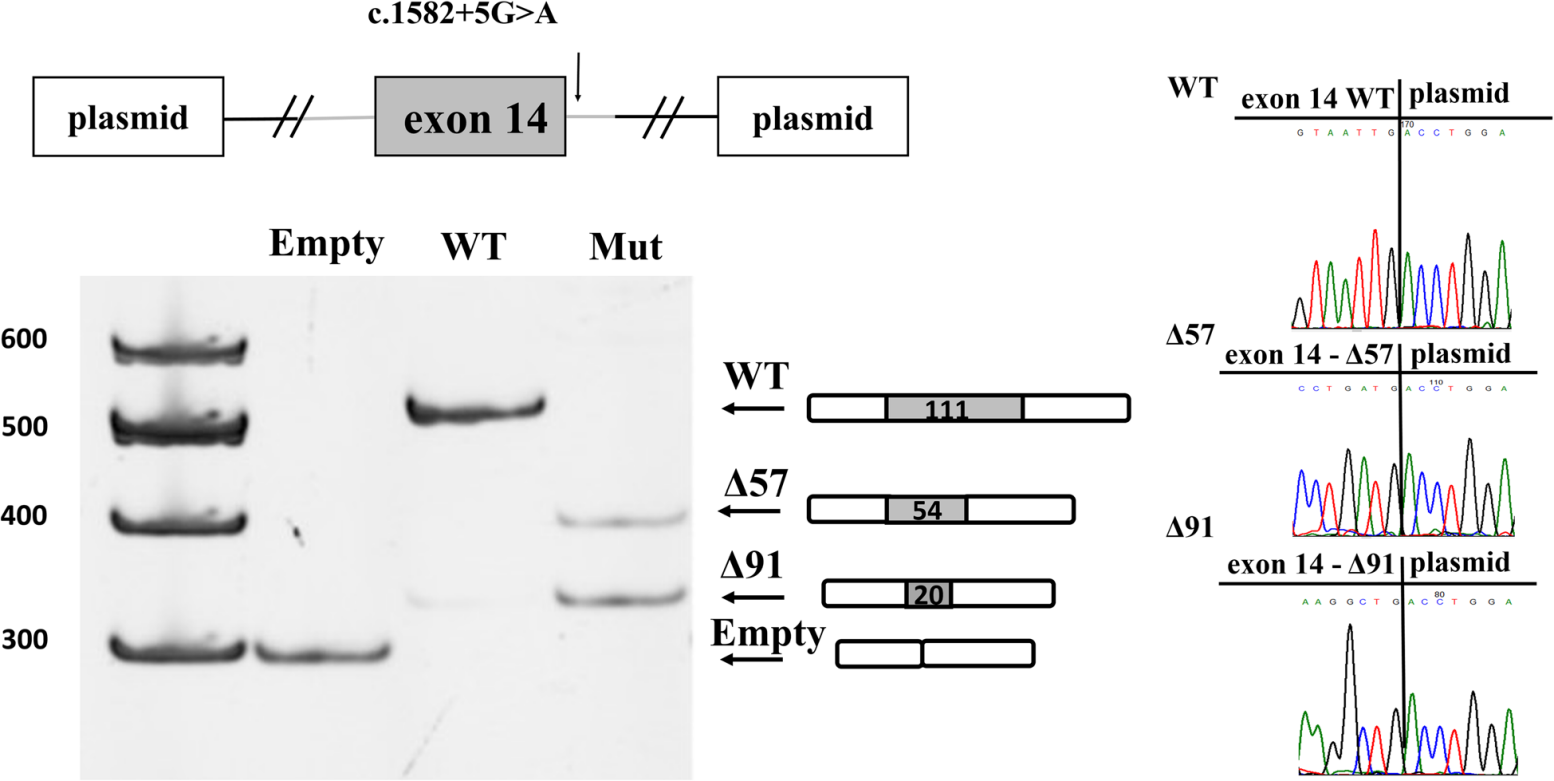
Functional analysis results. The minigene assay revealed that c.1582 + 5G > A variant leads to two independent pathogenic splicing alteration: a 57 (Δ57) and a 91 (Δ91) nucleotide truncation. On the top schematic minigene design is depicted. WT- wild type, Mut- mutation. The uncropped version of the gel image is available as additional file 1

In order to determine the impact of the described variant on splicing, we performed a minigene-splicing assay. We cloned the entire 14 exon with the flanking introniс sequences containing the variant of interest into pSpl3-Flu plasmid [8] and transfected HEK293T cells. Subsequent RT-PCR analysis with plasmid-specific primers showed that the presence of c.1582 + 5G > A variant lead to two independent pathogenic splicing alterations (Fig. 2). Sanger sequencing of RT-PCR products revealed that c.1582 + 5G > A variant disrupt the donor splice site (SS) of intron 14, which leads to activation of two upstream exonic cryptic SS. The activation of the first cryptic SS causes a 57-nucleotide truncation of exon 14 (Δ57) resulted in an in-frame deletion of 19 amino acids (p.(Gly509_ Ile527del)). These 19 amino acids encode for part of the fifteenth transmembrane α-helices of CLC-1 channel (helix O) and other pathogenic missense variants were described in this exon leading to MC [9, 10]. The second pathogenic splicing event is a 91-nucleotide truncation of exon 14 (Δ91) that leads to a frame-shift (p.(Val498GlufsTer5)) and a formation of a premature stop codon (PTC). Such mRNA isoform should be degraded by nonsense-mediated decay (NMD) [11]. According to functional investigation missense variants in exon 14 of the *CLCN1* gene are considered to be similar to loss of function (LoF) variants based on their effect on channel conductance [12]. This means that in our case both splicing alterations are predicted to cause LoF of CLC-1 protein. Thereby after performing functional study according to the ACMG guidelines we reclassify c.1582 + 5G > A variant as a class 4 – likely pathogenic (criteria PM2, PP3 and PS3).

## Discussion and conclusions

CLC-1 is the most abundant chloride channel expressed predominantly on the sarcolemma of skeletal muscles and plays a key role in excitability regulation. In human muscles, up to 80% of the resting membrane conductance is contributed by chloride conductance [13]. The chloride channels in muscle cells are activated after an action potential and are necessary for restoring the resting membrane potential. If the chloride channels fail to activate, K^+^ starts to accumulate in the extracellular space, which leads to membrane depolarization that at a certain level can initiate self-activation resulting in a prolonged muscle constriction that is observed in myotonia patients [14].

To date 280 pathogenic variants in *CLCN1* are described according to HGMD professional database and include missense, nonsense, splicing substitutions, and frameshift variants. Different pathogenic variants can lead to different forms of MC. The recessive form of MC is associated with biallelic LoF variants that can be found across all gene body, except for truncation variants located close to the C-terminus. Missense pathogenic variants can lead to both recessive and dominant MC. Dominant MС is associated with missense variants, and truncating variants, escaping NMD leading to a dominant-negative effect on CLC-1 homodimer channel [2].

Because of the diversity in the molecular bases of MC, it is rather difficult to establish the mode of inheritance based on the patient’s genotype and only functional investigations can resolve this issue. Moreover, functional studies increase our knowledge in understanding the pathogenesis of MC, which is crucial for more precise genetic counseling and developing future therapeutic approaches. Aberrant splicing is known to cause MC, however, it is very difficult to interpret variants that lay outside of the canonical SS dinucleotide. These intronic variants are often classified as VUS and evaluating their functional consequences is very important especially for families that consider procreation.

The most informative results for splicing analysis are obtained by using RT-PCR analysis from a disease-relevant tissue. Unfortunately, in many cases, it is not possible to perform such analysis for many reasons including medical and ethical issues. Nevertheless, several publications including the work of van der Klift et al. [15] showed that minigenes assays are highly concordant with patient RNA analysis in terms of rather or not the variant is pathogenic. In our case, the minigene assay revealed complete absence of the WT transcript and the presence of two aberrant isoforms. Further analysis of the mutated isoforms and their effect on the protein level led us to the conclusion that both transcripts should lead to a LoF which is the main pathogenic mechanism in recessive MC.

Here we present a clinical report of a proband with recessive form of MС in a consanguineous family with a variant affecting splicing in a homozygous state. This variant was reported as pathogenic before, however, no functional conformation was performed. Due to the fact that the proband mother was pregnant, we decided to functionally investigate this variant for performing proper prenatal diagnosis. By splicing minigene assay, we confirmed the pathogenic role of the splicing variant on the molecular level. At 10–11 weeks of gestation, a prenatal diagnosis was performed from chorion villi samples showing that the fetus is a heterozygous carrier of c.1582 + 5G > A variant.

## Supplementary information

**Additional file 1.** Uncropped version of the gel image in Fig. 2

## Data Availability

The datasets used and analysed during the current study are available from the corresponding author upon reasonable request. c.1582 + 5G > A variant was submitted to LOVD database. Variant ID #0000598290.

## Abbreviations

MC: Myotonia congenital
CLCN1: Chloride voltage-gated channel 1
LoF: Loss of function
VUS: Variant of uncertain significances
EMG: Electromyography
ACMG: American college of medical genetics
NMD: Nonsense-mediated decay
HGMD: Human gene mutation database
HGVS: Human genome variation society
OMIM: Online mendelian inheritance in man
