# Supplementary figures and images for: Recessive myotonia congenita caused by a homozygous splice site variant in CLCN1 gene: a case report
Source: BMC Med Genet. 2020 Oct 22;21(Suppl 1):197. doi: 10.1186/s12881-020-01128-5 (PMC7579786; doi:10.1186/s12881-020-01128-5)

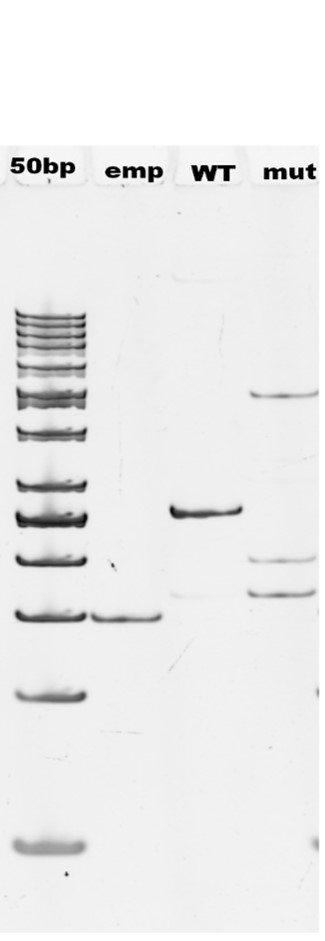

Supplement: Supplementary file 1 — Additional file 1. Uncropped version of the gel image in Fig. 2 [file 12881_2020_1128_MOESM1_ESM.jpg]
